# Heschl’s Gyrus Duplication Pattern in Individuals at Risk of Developing Psychosis and Patients With Schizophrenia

**DOI:** 10.3389/fnbeh.2021.647069

**Published:** 2021-04-20

**Authors:** Tsutomu Takahashi, Daiki Sasabayashi, Yoichiro Takayanagi, Yuko Higuchi, Yuko Mizukami, Shimako Nishiyama, Atsushi Furuichi, Mikio Kido, Tien Viet Pham, Haruko Kobayashi, Kyo Noguchi, Michio Suzuki

**Affiliations:** ^1^Department of Neuropsychiatry, Graduate School of Medicine and Pharmaceutical Sciences, University of Toyama, Toyama, Japan; ^2^Research Center for Idling Brain Science, University of Toyama, Toyama, Japan; ^3^Arisawabashi Hospital, Toyama, Japan; ^4^Health Administration Center, Faculty of Education and Research Promotion, Academic Assembly, University of Toyama, Toyama, Japan; ^5^Department of Radiology, Graduate School of Medicine and Pharmaceutical Sciences, University of Toyama, Toyama, Japan

**Keywords:** at-risk mental state, schizophrenia, Heschl’s gyrus, gyrification, early neurodevelopment

## Abstract

An increased prevalence of duplicated Heschl’s gyrus (HG), which may reflect an early neurodevelopmental pathology, has been reported in schizophrenia (Sz). However, it currently remains unclear whether individuals at risk of psychosis exhibit similar brain morphological characteristics. This magnetic resonance imaging study investigated the distribution of HG gyrification patterns [i.e., single HG, common stem duplication (CSD), and complete posterior duplication (CPD)] and their relationship with clinical characteristics in 57 individuals with an at-risk mental state (ARMS) [of whom 5 (8.8%) later developed Sz], 63 patients with Sz, and 61 healthy comparisons. The prevalence of duplicated HG patterns (i.e., CSD or CPD) bilaterally was significantly higher in the ARMS and Sz groups than in the controls, whereas no significant differences were observed in HG patterns between these groups. The left CSD pattern, particularly in the Sz group, was associated with a verbal fluency deficit. In the ARMS group, left CSD pattern was related to a more severe general psychopathology. The present results suggest that an altered gyrification pattern on the superior temporal plane reflects vulnerability factors associated with Sz, which may also contribute to the clinical features of high-risk individuals, even without the onset of psychosis.

## Introduction

Heschl’s gyrus (HG), a convolution on the superior temporal plane, hosts the primary auditory cortex (Rademacher et al., 1993; Da Costa et al., 2011) and is also involved in memory (Weinberger, 2015) and emotional (Concina et al., 2019) processing. The morphology of HG markedly varies across individuals, with approximately 30–50% of healthy individuals potentially having complete or partial duplication (Leonard et al., 1998; Rademacher et al., 2001; Abdul-Kareem and Sluming, 2008; Marie et al., 2015). This anatomical variant appears to reflect variations in cytoarchitectonic development during gestation (Chi et al., 1977; Armstrong et al., 1995), and duplicated HG may lead to learning disabilities (Leonard et al., 1993, 2001) and reduced HG activity during auditory processing (Tzourio-Mazoyer et al., 2015) in a non-clinical population. In a recent magnetic resonance imaging (MRI) study, we reported an increased prevalence of HG duplications in first-episode schizophrenia (Sz) (Takahashi et al., in submission), which may reflect the early neurodevelopmental pathology (Weinberger, 1987; Insel, 2010). However, since another MRI study that specifically examined HG duplication patterns in chronic Sz did not find significant results (Hubl et al., 2010), it currently remains unclear whether illness stages affect the HG pattern of Sz. Furthermore, although structural/functional abnormalities in the superior temporal plane may underlie the positive psychotic symptoms (Alderson-Day et al., 2015; Takahashi and Suzuki, 2018) as well as core trait abnormalities [e.g., deficits in social cognition (Mier et al., 2017) and verbal fluency (Antonova et al., 2004)] of Sz, it has not yet been clarified whether the HG gyrification pattern is associated with these clinical features.

MRI studies on individuals at high risk of developing psychosis [i.e., at-risk mental state (ARMS) (Yung et al., 2004, 2005)], who have an increased risk of developing psychosis within a short period of time [approximately 30% at 2 years (Fusar-Poli et al., 2012a)], generally showed similar gross morphological characteristics associated with early neurodevelopment [e.g., an altered sulcogyral pattern in the orbitofrontal region (Nakamura et al., 2019) and widespread cortical hypergyria (Sasabayashi et al., 2017)] to those of overt Sz. Since these brain anomalies are at least partly observed in participants without a later onset of psychosis (Sasabayashi et al., 2017; Nakamura et al., 2019), they may represent biological traits associated with general vulnerability to psychopathology. These gross brain characteristics may contribute to cognitive impairments in the Sz and ARMS groups (Takahashi et al., 2019a), supporting the presentation of cognitive impairments, particularly in social function (Lee et al., 2015) and verbal fluency (Fusar-Poli et al., 2012b), even before the onset of psychosis as a trait vulnerability marker. However, despite evidence of partly shared superior temporal gray matter reductions in the ARMS and Sz groups (Takahashi et al., 2010b), no MRI studies to date have specifically examined the HG duplication pattern and its potential contribution to clinical features (e.g., cognitive deficits) in the ARMS cohort.

Therefore, the present MRI study aimed to examine the HG gyrification pattern (single HG, partial duplication, and complete duplication) in ARMS individuals and Sz patients, compare it with those in healthy controls, and examine its potential contribution to clinical variables (symptoms, social and cognitive functions). Based on our previous MRI findings from an independent sample of Sz (Takahashi et al., in submission) as well as the potential role of brain gyrification as a stable neurodevelopmental marker (Chi et al., 1977; Armstrong et al., 1995), we predicted increased HG duplication in both the ARMS and Sz groups. We also speculated that the HG pattern in these groups may be associated with clinical variables that reflect trait abnormalities, such as cognitive impairments.

## Materials and Methods

### Participants

Fifty-seven ARMS individuals, 63 Sz patients, and 61 healthy controls participated in the present study (Table 1); they were physically healthy and had no history of severe obstetric complications, serious head trauma, neurological illness, substance abuse, or serious medical disease (e.g., diabetes, thyroid disease, hypertension, or steroid use). Handedness (Okada et al., 2014a), IQ scores measured using the Japanese version of the National Adult Reading Test (JART) (Matsuoka et al., 2006), and the personal and parental socioeconomic status (SES) (Okada et al., 2014b) were also evaluated. We recently detected an altered HG gyrification pattern in first-episode Sz (Takahashi et al., in submission); however, there was no sample overlap between these findings and the present results.

**TABLE 1:** Demographic/clinical characteristics and sociocognitive functions in ARMS, schizophrenia, and control subjects.

	**HC**	**ARMS**	**Sz**	**Group difference^a^**
	**(*N* = 61)**	**(*N* = 57)**	**(*N* = 63)**	
Male/female	32/29	34/23	29/34	Chi-square = 2.23, *p* = 0.329
Age	25.6 ± 3.2	18.6 ± 4.3	28.0 ± 9.4	*F*(2,178) = 34.93, *p* < 0.001; ARMS < HC, Sz
Height (cm)	166.0 ± 8.3	164.4 ± 9.0	163.2 ± 8.4	*F*(2,178) = 1.68, *p* = 0.190
Handedness (right/left/mixed)	40/6/15	35/5/17	52/2/9	Chi-square = 7.73, *p* = 0.102
Socioeconomic status	6.2 ± 0.9	3.2 ± 1.4	4.2 ± 1.4	*F*(2,178) = 92.20, *p* < 0.001; ARMS < Sz < HC
Parental socioeconomic status	5.9 ± 0.9	5.0 ± 0.9	4.8 ± 1.4	*F*(2,177) = 16.94, *p* < 0.001; ARMS, Sz < HC
Age at onset (years)	–	–	22.4 ± 7.4	–
Duration of illness (years)	–	–	5.5 ± 6.0	–
Dose of antipsychotics (HPD equiv., mg/day)	–	2.5 ± 1.8 (*N* = 14)	11.3 ± 7.8 (*N* = 51)	*F*(1,63) = 17.32, *p* < 0.001; ARMS < Sz
Type of antipsychotics (typical/atypical/mixed)	–	1/12/1	1/45/5	Fisher’s exact test, *p* = 0.585
Duration of antipsychotic medication (years)	–	0.7 ± 1.2 (*N* = 17)	5.2 ± 6.2 (*N* = 53)	*F*(1,68) = 8.78, *p* = 0.004; ARMS < Sz
Time between intake and onset (years)	–	1.5 ± 2.6 (*N* = 5)	–	–
PANSS				
Positive	–	11.6 ± 3.2	13.9 ± 5.6	*F*(1,118) = 7.45, *p* = 0.007; ARMS < Sz
Negative	–	15.3 ± 6.6	16.3 ± 5.6	*F*(1,118) = 0.63, *p* = 0.428
General	–	30.2 ± 7.9	31.0 ± 9.7	*F*(1,118) = 0.25, *p* = 0.619
mGAF psychological symptom		46.8 ± 11.2	44.7 ± 14.3	*F*(1,117) = 0.73, *p* = 0.395
mGAF social functioning	–	51.7 ± 10.2	48.2 ± 13.9	*F*(1,117) = 2.55, *p* = 0.113
SCoRS global rating score	–	5.3 ± 2.3	5.2 ± 2.5	*F*(1,117) = 0.02, *p* = 0.899
JART-IQ	110.2 ± 5.9	98.5 ± 9.7	99.5 ± 9.7	*F*(2,178) = 34.35, *p* < 0.001; ARMS, Sz < HC
BACS subdomain *z*-scores				Group × domain interaction, *F*(5,590) = 6.29, *p* < 0.001
Verbal memory	–	−0.7 ± 1.6	−1.4 ± 1.4	*p* = 0.347
Working memory	–	−0.7 ± 1.3	−1.0 ± 1.4	*p* = 1.000
Motor function	–	−0.9 ± 1.4	−1.9 ± 1.5	*p* = 0.004; Sz < ARMS
Verbal fluency	–	−0.9 ± 1.5	−0.8 ± 1.1	*p* = 1.000
Attention and processing speed	–	−0.2 ± 1.4	−1.4 ± 1.5	*p* < 0.001; Sz < ARMS
Executive function	–	−0.3 ± 1.2	−0.8 ± 1.6	*p* = 0.840

*Values represent means ± SD unless otherwise stated.ARMS, at risk mental state; BACS, Brief Assessment of Cognition in Schizophrenia; HC, healthy controls; JART, Japanese version of National Adult Reading Test; HPD, haloperidol; mGAF, modified Global Assessment of Functioning; PANSS, Positive and Negative Syndrome Scale; SCoRS, Schizophrenia Cognition Rating Scale; Sz, schizophrenia.^*a*^Differences between the degree of freedom across measures were partly attributed to missing data.*

As described previously (Takahashi et al., 2017, 2018), individuals with ARMS were enrolled from the Consultation Support Service in Toyama (CAST), which is a regional clinical setting that specializes in early interventions (Mizuno et al., 2009). All individuals met the criteria for attenuated psychotic symptoms (APS) based on the Comprehensive Assessment of At-Risk Mental States (CAARMS) (Yung et al., 2005), while 6 also fulfilled brief and limited intermittent psychotic symptoms (BLIPS) (*N* = 1) or genetic risk and deterioration syndrome (GRD) (*N* = 5) criteria. Major comorbid DSM Axis I disorder (American Psychiatric Association, 2000) comprised anxiety disorders (*N* = 13), adjustment disorders (*N* = 11), schizotypal personality disorders (*N* = 10), pervasive developmental disorders (*N* = 9), or depressive disorders (*N* = 8). Five participants (8.8%) developed Sz during the clinical follow-up at Toyama University Hospital (mean = 3.2 ± 2.9 years, median = 2.4). Medication and other clinical data are summarized in Table 1. Eleven participants were also being treated with antidepressants (*N* = 5) and/or benzodiazepines (*N* = 8) when scans were performed.

Sz patients fulfilling the DSM-IV-TR criteria (American Psychiatric Association, 2000) were enrolled from the in- and outpatient clinics of the Department of Neuropsychiatry of Toyama University Hospital. They were diagnosed based on the Structured Clinical Interview for DSM-IV Axis I Disorders Patient Edition (SCID-I/P) (First et al., 1997) and a detailed chart review. The Sz group was divided into first-episode [illness duration ≤1 year (*N* = 17)] and chronic [illness duration ≥3 years (*N* = 38)] subgroups to examine the effects of illness chronicity.

Healthy controls with no personal or family history (among first-degree relatives) of neuropsychiatric disorders were enrolled from both the community and hospital staff and screened using the SCID-I Non-patient Edition (First et al., 1997). The present study was approved by the Committee on Medical Ethics of Toyama University (No. I2013006). Written informed consent was obtained from all participants in accordance with the Declaration of Helsinki. When participants were <20 years old, written consent was also obtained from a parent/guardian.

### Clinical Assessment at Scanning

The clinical symptoms of ARMS and Sz participants were rated by experienced psychiatrists using the Positive and Negative Syndrome Scale (PANSS) (Kay et al., 1987). The Brief Assessment of Cognition in Schizophrenia (BACS) (Keefe et al., 2004), the Schizophrenia Cognition Rating Scale (SCoRS) (Keefe et al., 2006), and the modified Global Assessment of Functioning (mGAF) scale (Eguchi et al., 2015) were used to evaluate social and cognitive functions.

### MRI Acquisition and Data Processing

Magnetic resonance imaging was performed using the 3-T Magnetom Verio (Siemens, Erlangen, Germany). A three-dimensional magnetization-prepared rapid gradient echo (MPRAGE) sequence provided 176 contiguous 1.2-mm-thick T1-weighted slices in the sagittal plane. The following imaging parameters were used: repetition time = 2,300 ms; echo time = 2.9 ms; flip angle = 9°; field of view = 256 mm; and matrix size = 256 pixels × 256 pixels, with a voxel size of 1.0 mm × 1.0 mm × 1.2 mm.

Brain images were coded randomly and analyzed blind to participants’ information (e.g., diagnosis and gender). The images were then realigned using Dr. View software (Infocom, Tokyo, Japan) into three dimensions to account for differences in head tilting during the acquisition of images. They were reconstructed into entire contiguous 1-mm-thick coronal images that were perpendicular to the anterior commissure-posterior commissure line.

### Assessment of HG Gyrification Patterns

**FIGURE 1:**
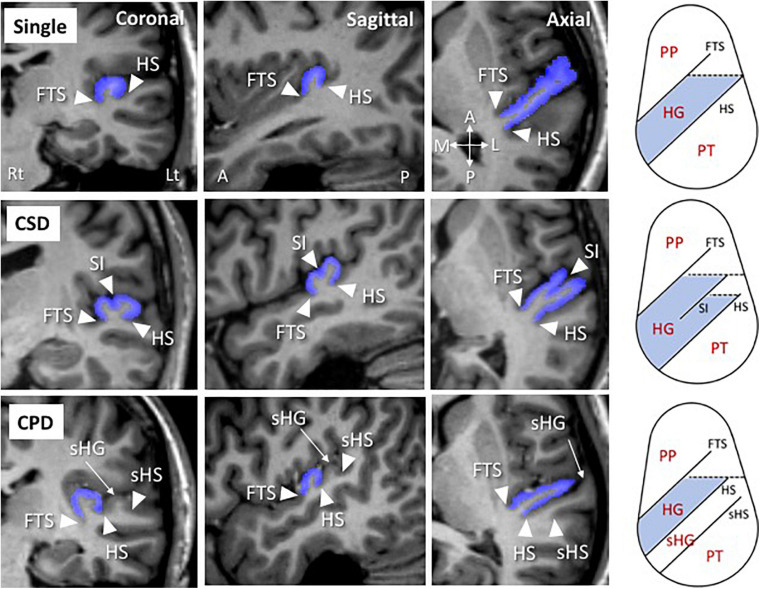
Sample MR images of Heschl’s gyrus (HG; colored in blue) in participants with different gyrification patterns. Schematic drawings of the superior temporal surface on an axial view are also shown (right). A, anterior; CPD, complete posterior duplication; CSD, common stem duplication; FTS, first transverse sulcus; HS, Heschl’s sulcus; L, lateral; Lt, left; P, posterior; M, medial; PP, planum polare; PT, planum temporale; Rt, right; sHG, second Heschl’s gyrus; sHS, second Heschl’s sulcus; SI, sulcus intermedius.

As reported previously (Leonard et al., 1998; Rademacher et al., 2001; Abdul-Kareem and Sluming, 2008; Marie et al., 2015), the HG gyrification pattern on each hemisphere was classified into single HG, common stem duplication (CSD), and complete posterior duplication (CPD) (Figure 1). Among duplicated HG patterns, the CSD pattern was characterized by the gyrus being partially split by the sulcus intermedius (SI), which forms a ‘heart-shaped’ HG. The hemisphere with fully separate gyri [two (*N* = 80) or three (*N* = 4) gyri per hemisphere in the present study] was defined as the CPD pattern. Fourteen hemispheres (3.9%), which had a separate HG posterior to the HG with partial duplication, were categorized as the CSD pattern.

In the present study, one rater (TT) classified HG gyrification patterns without knowledge of subject identities. Intra- (TT) and inter-rater (TT and DS) reliabilities in 15 randomly selected brains (30 hemispheres) were ≥0.83 (Cronbach’s α).

### Statistical Analysis

Demographic and clinical data were compared between groups using a one-way analysis of variance (ANOVA) or the χ^2^ test.

Group differences in the HG pattern distribution were compared on each hemisphere by the χ^2^ test. Potential relationship between the HG pattern and age, IQ, or medication (dose, duration) was assessed using ANOVA with the HG pattern as an independent variable. For assessing the potential contribution of the HG pattern to clinical variables (PANSS, BACS, SCoRS, and mGAF scores) in the ARMS and Sz groups, analysis of covariance (ANCOVA) was used with age and medication (dose, duration) as covariates. The relationship between the HG pattern and clinical variables with non-normal distribution (SCoRS, mGAF, and BACS executive function scores for both groups and BACS verbal/working memory scores for Sz group; tested by Kolmogorov–Smirnov tests) was also assessed by non-parametric Kruskal–Wallis tests. PANSS and other BACS scores were normally distributed. A *post hoc* Newman–Keuls test was used to follow-up these analyses. A *p*-value of <0.05 was considered to be significant.

## Results

### Demographic and Clinical Characteristics (Table 1)

No significant differences were observed in sex, height, or handedness between groups, whereas age, IQ, and parental/personal SES significantly differed.

Lower doses of antipsychotics, less severe positive symptoms, and higher BACS scores for motor function and attention subdomains were observed in the ARMS group than in the Sz group.

### HG Pattern Distributions

**FIGURE 2:**
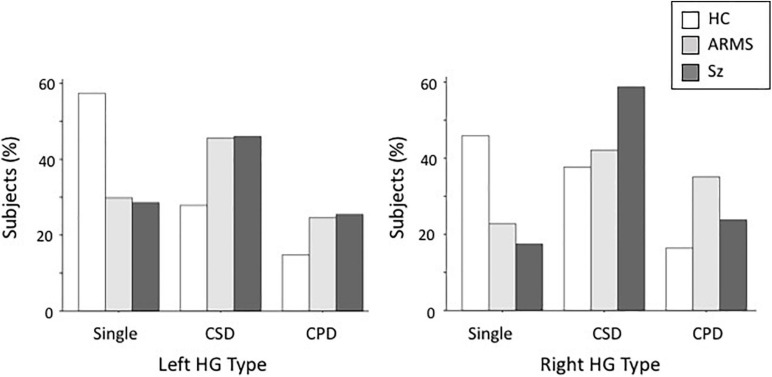
Distribution of Heschl’s gyrus (HG) gyrification patterns in schizophrenia (SZ), at-risk mental state (ARMS), and healthy comparison (HC) groups. CPD, complete posterior duplication; CSD, common stem duplication.

Both the ARMS (left, χ^2^ = 9.08, *p* = 0.003; right, χ^2^ = 6.93, *p* = 0.008) and Sz (left, χ^2^ = 10.51, *p* = 0.001; right, χ^2^ = 11.63, *p* < 0.001) groups had a significantly higher prevalence of duplicated HG patterns (i.e., CSD or CPD) bilaterally than the controls, whereas the HG pattern did not significantly differ between these groups (left, χ^2^ = 0.02, *p* = 0.880; right, χ^2^ = 0.53, *p* = 0.465) (Table 2 and Figure 2). When we examined participants with HG duplication only, no group difference was noted in HG patterns (CSD vs. CPD; all χ^2^ < 1.82, *p* > 0.177). We also compared the first-episode and chronic subgroups of Sz, but found no significant differences in the HG patterns (left, χ^2^ = 0.60, *p* = 0.741; right, χ^2^ = 0.06, *p* = 0.969).

**TABLE 2:** Gyrification pattern of Heschl’s gyrus (HG) in study participants.

**Healthy controls**					
		**Right HG pattern [*N* (%)]**			
		**Single**	**CSD**	**CPD**	**Total**

Left HG pattern [*N* (%)]	Single	17 (27.9)	11 (18.0)	7 (11.5)	35 (57.4)
	CSD	7 (11.5)	8 (13.1)	2 (3.3)	17 (27.9)
	CPD	4 (6.6)	4 (6.6)	1 (1.6)	9 (14.8)
	Total	28 (45.9)	23 (37.7)	10 (16.4)	61 (100.0)

**ARMS**					

		**Right HG pattern [*N* (%)]**			
		**Single**	**CSD**	**CPD**	**Total**

Left HG pattern [*N* (%)]	Single	4 (7.0)	7 (12.3)	6 (10.5)	17 (29.8)
	CSD	8 (14.0)	11 (19.3)	7 (12.3)	26 (45.6)
	CPD	1 (1.8)	6 (10.5)	7 (12.3)	14 (24.6)
	Total	13 (22.8)	24 (42.1)	20 (35.1)	57 (100.0)

**Schizophrenia**					

		**Right HG pattern [*N* (%)]**			
		**Single**	**CSD**	**CPD**	**Total**

Left HG pattern [*N* (%)]	Single	7 (11.1)	10 (15.9)	1 (1.6)	18 (28.6)
	CSD	2 (3.2)	19 (30.2)	8 (12.7)	29 (46.0)
	CPD	2 (3.2)	8 (12.7)	6 (9.5)	16 (25.4)
	Total	11 (17.5)	37 (58.7)	15 (23.8)	63 (100.0)

*CSD, common stem duplication; CPD, complete posterior duplication.*

Furthermore, HG patterns did not significantly differ between male and female participants (left, χ^2^ = 0.87, *p* = 0.648; right, χ^2^ = 1.03, *p* = 0.596), while HG duplication (i.e., CSD or CPD) was more frequent in the right hemisphere (χ^2^ = 4.01, *p* = 0.045) when all diagnostic groups were combined.

### HG Pattern and Clinical Variables

Medication (for the ARMS and Sz groups), age, and IQ were not associated with the HG pattern for all diagnostic groups (Supplementary Table).

In the combined sample of ARMS and Sz participants, there was a significant effect of the left HG pattern on the BACS verbal fluency score [*F*(2,114) = 3.89, *p* = 0.023]; participants with CSD had a lower score than those with CPD (*p* = 0.040). This effect was significant also for the Sz group only [*F*(2,57) = 3.69, *p* = 0.031; *post hoc* test, *p* = 0.044].

At-risk mental state individuals with the left CSD pattern had a higher PANSS general psychopathology score than those with the CPD pattern [*F*(2,51) = 4.97, *p* = 0.011; *post hoc* test, *p* = 0.016].

No association was observed between the HG pattern and other clinical variables (e.g., SCoRS and mGAF scores; Supplementary Table).Kruskal–Wallis tests for the clinical variables with non-normal distribution also showed no significant association.

## Discussion

To the best of our knowledge, this is the first MRI study to examine the HG duplication pattern in clinical high-risk individuals for developing psychosis. We demonstrated that ARMS individuals and patients with established Sz both exhibited a significantly higher prevalence of duplicated HG patterns than healthy controls. Furthermore, the HG pattern was associated with global symptom ratings and verbal fluency ability in these participants. The present results suggest that the gross morphological characteristics of the superior temporal plane represent vulnerability factors associated with psychosis, which may be associated with clinical trait abnormalities.

The present study replicated our previous findings from an independent cohort of first-episode Sz (Takahashi et al., in submission) showing increased HG duplication in Sz patients and also demonstrated that illness stages (i.e., first-episode vs. chronic stages) did not significantly influence HG patterns. On the other hand, a previous study by Hubl et al. (2010) only found a slightly higher prevalence of duplicated HG in chronic Sz patients. However, their negative finding may be partly due to the small sample size examined (13 Sz and 13 control participants) as well as their definition of HG duplication, which classified the CSD pattern as a variant of single HG. Since we demonstrated increased HG duplication in Sz regardless of the subtype (i.e., CSD or CPD), the Sz group examined by Hubl et al. (2010) must have had a higher prevalence of the duplicated HG pattern according to the traditional HG pattern definition [single vs. duplicated (CSD or CPD) (Leonard et al., 1998; Rademacher et al., 2001; Abdul-Kareem and Sluming, 2008; Marie et al., 2015)]. While the mechanisms regulating the development of cortical gyrification remain unclear, the secondary gyri of HG, which form variations in the HG gyrification pattern, predominantly develop during the late gestation period (i.e., after 36 weeks of gestation) (Chi et al., 1977) along with local neuronal connectivity and synaptic development (Van Essen, 1997), but remain stable after birth (Armstrong et al., 1995). Therefore, HG gyrification studies in Sz generally support the notion that the gyrification pattern in Sz represents a stable trait marker associated with early neurodevelopmental pathology (Matsuda and Ohi, 2018).

One of the primary results of the present study was that ARMS individuals, who may be vulnerable to psychopathology but will not necessarily develop overt psychosis (Yung et al., 2004; Fusar-Poli et al., 2012a), exhibited an increased HG duplication pattern similar to that in Sz. Based on the potential relationship between brain gyrification and local neuronal connectivity (Van Essen, 1997), the present results appear to be consistent with previous functional neuroimaging findings showing that the ARMS and Sz groups share local connectivity disruption involved in HG (Yoon et al., 2015; Du et al., 2018). A few MRI studies on cortical surface features in clinical high-risk individuals also showed similar gross morphological characteristics, such as altered sulcogyral patterns (Sasabayashi et al., 2017; Nakamura et al., 2019) and sulcal-depth abnormalities (Takahashi et al., 2019b), with patients with established Sz. In contrast to the evidence of active gray matter reductions in the superior temporal plane (e.g., HG and planum temporale) during the early illness stages of psychosis (Takahashi and Suzuki, 2018), a recent longitudinal study demonstrated the stability of gyrification features during the clinical high-risk period as a marker of early neurodevelopmental insults (Damme et al., 2019). Nevertheless, high-risk individuals with the later onset of psychosis may exhibit greater gyrification abnormalities before illness onset (Sasabayashi et al., 2017; Das et al., 2018) because greater and/or more prolonged neurodevelopmental deviations during gestation and consequent anomalous post-pubertal brain changes may lead to overt and sustained psychosis (Pantelis et al., 2005). Since the present ARMS group with a short follow-up period (median = 2.4 years) only examined a small number of participants with a later onset of psychosis (*N* = 5), the potential of the HG gyrification pattern as a predictive marker of the later onset of psychosis remains unclear.

The present results suggested that the partial duplication of HG (i.e., CSD) was associated with a more severe general psychopathology in ARMS individuals, supporting aberrant connectivity in the superior temporal region potentially contributing to prodromal-like symptoms (Yoon et al., 2015). However, the present Sz cohort (predominantly chronic cases) did not replicate the relationship between the CPD pattern and mild positive symptom severity observed in first-episode Sz (*N* = 62) (Takahashi et al., in submission), implicating that neurodevelopmental pathology may be associated with susceptibility to positive psychotic symptoms of Sz but this relationship may be influenced by various factors including illness stages and treatment. On the other hand, as also suggested in our sample (Table 1), cognitive deficits, particularly in verbal fluency and memory functioning, may exist even before the onset of psychosis as markers of increased vulnerability (Fusar-Poli et al., 2012b; Lee et al., 2015). In the present study, we found that participants with the left CSD pattern had a greater deficit in verbal fluency, but not in other domains or social functioning, than those with the left CPD pattern in the Sz (*N* = 63) or combined Sz and ARMS (*N* = 120) groups. This result appears to be consistent with the notion that candidate neural circuits for verbal fluency deficits include the superior temporal region for both the Sz (Frith et al., 1995; Antonova et al., 2004) and ARMS (Meijer et al., 2011) groups. While the functional role of the HG duplication type (i.e., CPD vs. CSD) remains largely unknown, participants with the CSD pattern may have a significantly smaller planum temporale gray matter than those with the CPD pattern bilaterally for both the Sz and control groups (Takahashi et al., in submission), which may lead to deficits in verbal ability (Shapleske et al., 1999). However, the potential contribution of different HG patterns to the pathophysiology of psychotic disorders warrants further study at various illness stages, particularly using functional neuroimaging.

Several potential limitations in the present study need to be addressed. First, as described above, it was not possible to examine whether the HG gyrification pattern was associated with the future onset of psychosis because only 5 participants (8.8%) in the ARMS group developed psychosis in the clinical follow-up period. Furthermore, the ARMS group was younger than the other groups in the present study. Second, the majority of Sz and 14 ARMS participants were being treated with antipsychotics during the present study. These factors were not expected to significantly affect gross sulcogyral patterns; however, antipsychotic medication may be a confounding factor for the morphology of the superior temporal plane (Takahashi and Suzuki, 2018) and cognitive functioning (Keefe, 2014). Therefore, future studies using a larger antipsychotic naïve ARMS cohort (particularly participants with a later onset of psychosis) and well-matched comparison groups are needed to examine the HG gyrification pattern and its potential contribution to clinical features (including the later onset of psychosis). Third, we did not correct our results of ANOVA/ANCOVA for multiple comparisons due to exploratory nature of our study. We predicted that the HG pattern would be associated with cognitive impairments, but we had no clear hypothesis and comprehensively assessed the potential contribution of HG pattern to all available cognitive subdomains, which might lead to potential Type I error. Finally, since superior temporal gray matter reductions (Takahashi et al., 2010a, c) and altered brain gyrification patterns (Yang et al., 2016; Maggioni et al., 2019) have been reported in other neuropsychiatric disorders (e.g., mood and anxiety disorders and autism), the disease specificity of the present results warrant further study.

## Conclusion

The results of this MRI study demonstrated that clinical high-risk individuals for psychosis exhibited an increased HG duplication similar to that in patients with Sz, which may reflect common vulnerability factors. These groups partly shared cognitive impairments, which were associated with HG gyrification patterns. We also found a relationship between the HG pattern and severity of general symptoms observed in high-risk individuals. Therefore, the gross morphology of the superior temporal plane may represent the biological trait abnormalities of Sz that exist prior to illness onset; however, our findings should be replicated in an independent and larger cohort especially for high-risk individuals with and without the later onset of psychosis in order to investigate potential role of HG pattern as a predictive marker of Sz.

## Data Availability Statement

The data analyzed in this study is subject to the following licenses/restrictions: the datasets generated during the current study will not be available for public use, since we do not have permission to share the data. Requests to access these datasets should be directed to TT, tsutomu@med.u-toyama.ac.

## Ethics Statement

The studies involving human participants were reviewed and approved by the Committee on Medical Ethics of Toyama University. Written informed consent to participate in this study was provided by the participants’ legal guardian/next of kin.

## Author Contributions

MS, YH, and TT conceived the idea and methodology of the study. TT conducted the statistical analyses and wrote the manuscript. DS, YH, MK, and HK recruited participants and were involved in clinical and diagnostic assessments. TT, DS, and TP analyzed MRI data. YM and SN assessed the sociocognitive functions of the study participants. KN provided technical support for MRI scanning and data processing. AF managed the MRI and clinical data. MS and YT contributed to the writing and editing of the manuscript. All authors contributed to and approved the final manuscript.

## Conflict of Interest

The authors declare that the research was conducted in the absence of any commercial or financial relationships that could be construed as a potential conflict of interest.
