# Supplementary material for: Heschl’s Gyrus Duplication Pattern in Individuals at Risk of Developing Psychosis and Patients With Schizophrenia
Source: Front Behav Neurosci. 2021 Apr 20;15:647069. doi: 10.3389/fnbeh.2021.647069 (PMC8093503; doi:10.3389/fnbeh.2021.647069)
Supplement: Supplementary file 1 [file Table_1.DOCX]

Supplementary Table. ANOVA/ANCOVA results of the relationship between demographic/clinical data and Heschl's gyrification pattern in the study participants.

	Whole sample (N = 181)		Healthy controls (N = 61)		ARMS + Sz (N = 120)		ARMS (N = 57)		Sz (N = 63)	
	Left	Right	Left	Right	Left	Right	Left	Right	Left	Right
Age	F (2, 178) = 0.43, p = 0.652	F (2, 178) = 0.45, p = 0.637	F (2, 58) = 0.78, p = 0.461	F (2, 58) = 0.05, p = 0.956	F (2, 117) = 0.50, p = 0.610	F (2, 117) = 0.69, p = 0.504	F (2, 54) = 0.05, p = 0.955	F (2, 54) = 0.10, p = 0.909	F (2, 60) = 1.01, p = 0.369	F (2, 60) = 3.09, p = 0.053
JART-IQ	F (2, 178) = 1.38, p = 0.255	F (2, 178) = 0.76, p = 0.471	F (2, 58) = 0.08, p = 0.920	F (2, 58) = 1.46, p = 0.240	F (2, 117) = 1.12, p = 0.331	F (2,117) = 0.87, p = 0.421	F (2, 54) = 1.17, p = 0.319	F (2, 54) = 1.52, p = 0.228	F (2, 60) = 0.32, p = 0.731	F (2, 60) = 0.04, p = 0.960
Medication dose	-	-	-	-	F (2, 117) = 0.45, p = 0.640	F (2, 117) = 1.52, p = 0.222	F (2 ,54) = 0.59, p = 0.559	F (2, 54) = 0.21, p = 0.814	F (2, 60) = 1.06, p = 0.354	F (2, 60) = 3.12, p = 0.052
Medication duration	-	-	-	-	F (2, 117) = 0.75, p = 0.474	F (2,117) = 1.43, p = 0.244	F (2, 54) = 0.13, p = 0.881	F (2, 54) = 0.28, p = 0.755	F (2, 60) = 1.12, p = 0.334	F (2, 60) = 1.49, p = 0.369
PANSS positive	-	-	-	-	F (2, 114) = 0.48, p = 0.622	F (2,117) = 0.53, p = 0.590	F (2, 51) = 0.36, p = 0.700	F (2, 51) = 0.95, p = 0.392	F (2, 57) = 1.44, p = 0.246	F (2, 57) = 0.79, p = 0.459
PANSS negative	-	-	-	-	F (2, 114) = 0.21, p = 0.811	F (2, 114) = 0.42, p = 0.658	F (2, 51) = 1.83, p = 0.170	F (2, 51) = 0.17, p = 0.847	F (2, 57) = 1.96, p = 0.150	F (2, 57) = 0.32, p = 0.729
PANSS general	-	-	-	-	F (2, 114) = 0.14, p = 0.869	F (2, 114) = 0.71, p = 0.494	**F (2, 51) = 4.97, p = 0.011**	F (2, 51) = 0.28, p = 0.759	F (2, 57) = 2.26, p = 0.113	F (2, 57) = 1.28, p = 0.286
mGAF symptom	-	-	-	-	F (2, 113) = 0.59, p = 0.555	F (2, 113) = 1.19, p = 0.310	F (2, 51) = 3.06, p = 0.055	F (2, 51) = 2.86, p = 0.067	F (2, 56) = 0.14, p = 0.873	F (2, 56) = 0.23, p = 0.793
mGAF functioning	-	-	-	-	F (2, 113) = 1.36, p = 0.261	F (2, 113) = 1.25, p = 0.289	F (2, 51) = 0.97, p = 0.388	F (2, 51) = 2.13, p = 0.130	F (2, 56) = 0.58, p = 0.565	F (2, 56) = 0.03, p = 0.971
SCoRS	-	-	-	-	F (2, 113) = 1.60, p = 0.206	F (2, 113) = 0.36, p = 0.697	F (2, 51) = 2.27, p = 0.114	F (2, 51) = 0.89, p = 0.419	F (2, 56) = 1.04, p = 0.362	F (2, 56) = 0.38, p = 0.687
BACS verbal memory	-	-	-	-	F (2, 114) = 1.95, p = 0.147	F (2, 114) = 0.60, p = 0.553	F (2, 51) = 1.02, p = 0.367	F (2, 51) = 0.04, p = 0.966	F (2, 57) = 1.51, p = 0.231	F (2, 57) = 1.89, p = 0.160
BACS working memory	-	-	-	-	F (2, 114) = 0.29, p = 0.749	F (2, 114) = 0.85, p = 0.429	F (2, 51) = 0.03, p = 0.970	F (2, 51) = 2.69, p = 0.077	F (2, 57) = 0.54, p = 0.588	F (2, 57) = 0.74, p = 0.480
BACS motor function	-	-	-	-	F (2, 114) = 0.87, p = 0.421	F (2, 114) = 0.18, p = 0.835	F (2, 51) = 0.24, p = 0.785	F (2, 51) = 0.17, p = 0.842	F (2, 57) = 0.90, p = 0.413	F (2, 57) = 0.38, p = 0.684
BACS verbal fluency	-	-	-	-	**F (2, 114) = 3.89, p = 0.023**	F (2, 114) = 1.49, p = 0.230	F (2, 51) = 1.36, p = 0.265	F (2, 51) = 1.38, p = 0.261	**F (2, 57) = 3.69, p = 0.031**	F (2, 57) = 0.10, p = 0.905
BACS attention	-	-	-	-	F (2, 114) = 0.31, p = 0.734	F (2, 114) = 1.94, p = 0.148	F (2, 51) = 0.80, p = 0.453	F (2, 51) = 1.43, p = 0.249	F (2, 57) = 0.93, p = 0.401	F (2, 57) = 0.41, p = 0.668
BACS executive function	-	-	-	-	F (2, 114) = 1.63, p = 0.201	F (2, 114) = 0.29, p = 0.746	F (2, 51) = 1.69, p = 0.196	F (2, 51) = 0.13, p = 0.876	F (2, 57) = 0.46, p = 0.637	F (2, 57) = 0.25, p = 0.782

ANCOVA, analysis of covariance; ANOVA, analysis of variance; ARMS, at risk mental state; BACS, Brief Assessment of Cognition in Schizophrenia; JART, Japanese version of National Adult Reading Test; mGAF, modified Global Assessment of Functioning; PANSS, Positive and Negative Syndrome Scale; SCoRS, Schizophrenia Cognition Rating Scale; Sz, schizophrenia.
